# FIGO recommendations on objective measurement of blood loss after birth for early detection of postpartum hemorrhage

**DOI:** 10.1002/ijgo.70523

**Published:** 2025-09-23

**Authors:** Ferdousi Begum, Albaro J. Nieto‐Calvache, Dietmar Schlembach, Justus Hofmyer, Jose Palacios‐Jaraquemada, Ajey Bhardwaj, Maria A. Suarez, Juan M. Burgos‐Luna, Jolly Beyeza‐Kashesya, Akaninyene E. Ubom, Alison Wright, FIGO Committee on Childbirth and PPH

**Affiliations:** ^1^ Institute of Woman and Child Health Dhaka Bangladesh; ^2^ OGSB Hospital and Institute of Reproduction and Child Health Dhaka Bangladesh; ^3^ Departamento de Ginecología y Obstetricia Fundación Valle del Lili Cali Colombia; ^4^ Faculty of Health Sciences, Universidad ICESI Cali Colombia; ^5^ Vivantes Clinics Berlin‐Neukölln Berlin Germany; ^6^ Effective Care Research Unit University of the Witwatersrand and Walter Sisulu University East London South Africa; ^7^ University of Botswana Gaborone Botswana; ^8^ Hospital Universitario CEMIC Buenos Aires Argentina; ^9^ Airlangga University East Java Indonesia; ^10^ Avni Health Foundation Mumbai India; ^11^ Centro De Investigaciones Clínicas Fundación Valle Del Lili Cali Colombia; ^12^ Mulago Specialised Women and Neonatal Hospital Kampala Uganda; ^13^ World Association of Trainees in Obstetrics & Gynecology (WATOG) Paris France; ^14^ Royal Free Hospital London UK; ^15^ University College London London UK; ^16^ FIGO London UK

**Keywords:** blood loss, evidence‐based practice, health resources, maternal mortality, measurement, multiprofessional working, postpartum hemorrhage, surgical, teamwork

## Abstract

Postpartum hemorrhage (PPH) remains the leading cause of maternal mortality worldwide, particularly in resource‐constrained and remote settings. The cornerstone of reducing PPH‐related morbidity and mortality lies in its early recognition, timely treatment, and adherence to evidence‐based protocols, all of which are heavily dependent on an accurate assessment of postpartum blood loss. Visual estimation, unfortunately, is still widely used, highly inaccurate, and often leads to underdiagnosis, resulting in a delayed or sometimes absent response to PPH. Objective quantification of blood loss, although not perfectly precise, provides a far more reliable estimate and is critical to triggering timely and effective interventions. This approach requires coordinated teamwork, leadership, institutional commitment, and a shift in clinical culture from subjective to standardized measurement practices. Scientific evidence strongly supports the integration of objective quantification into routine obstetric care. The use of calibrated drapes, cumulative measurement, and the combination of quantification with early warning systems have all proven to be effective in reducing PPH‐related complications and deaths. Practical barriers are faced in implementing objective measurement strategies at the facility and institutional level, including availability, supply costs, environmental concerns, national and institutional advocacy, policy change, leadership, appropriate training, and, more importantly, resistance to change. Low‐cost, reusable, and locally made devices can offer promising solutions for scaling up this intervention in resource‐limited environments. Sustained success will depend on engagement with health departments of the governments for framing policies, guidelines for implementation, procurement and regular supplies, inter‐institutional collaboration, and ongoing and refresher training for in‐service healthcare providers. In the longer term, it requires inclusion into the medical curriculum, local leadership, champions, behavioral interventions, recognition and rewards to individuals, and teams who promote long‐term adherence to best practice. Ultimately, although technical knowledge on PPH management is now well established, the real challenge lies in its consistent and context‐appropriate identification and application. This article discusses the different definitions of PPH. It highlights the importance of objective quantification of blood loss during and after childbirth and the various available methods for blood loss measurement and explores their implementation across different clinical settings.

## 1 INTRODUCTION

Each year, millions of women experience postpartum hemorrhage (PPH), resulting in approximately 70 000 maternal deaths globally, which is approximately 20% of all maternal deaths. Most of these occur in resource‐limited settings^1^ and are almost entirely preventable.^2^ Multiple effective interventions exist for the management of PPH, applicable even at basic levels of care provision; however, delays in recognizing and diagnosing the condition are common, leading to late or non‐utilization of these interventions.^3^

There are various definitions of PPH, with the current definition proposed by the World Health Organization (WHO) being the most widely used.^4^ WHO currently defines PPH as blood loss exceeding 500 mL after a vaginal delivery or 1000 mL after a cesarean birth^4^, ^5^ within the first 24 h postpartum (see Box 1).

BOX 1 **Definition of PPH.**

**Table:** 

**Lack of consensus on definition** No single, universally accepted definition of PPH exists Definitions vary in thresholds and physiological criteria Precise volume‐based definitions are preferable **ACOG and WHO Definitions** **ACOG:** ≥1000 mL blood loss or signs of hypovolemia within 24 h of birth, regardless of delivery mode **WHO:** ≥500 mL for vaginal birth; ≥1000 mL for cesarean birth, acknowledging resource limitations and estimation challenges **Toward lower thresholds** Experts increasingly recommend **earlier intervention**; for example: ⚬ 500 mL with ongoing bleeding during cesarean ⚬ 300 mL + clinical sign after vaginal birth (E‐MOTIVE study) Lower thresholds aim to reduce delays and improve outcomes **Alternative clinical criteria** Use of surrogate indicators when blood loss cannot be quantified: ⚬ Hematocrit drop >10% ⚬ Hemodynamic instability ⚬ Lower blood loss volumes are needed as threhold if the patient has intolerance to blood loss due to pre‐existing conditions

Abbreviation: ACOG, American College of Obstetricians and Gynecologists; PPH, postpartum hemorrhage; WHO, World Health Organization.

Visual estimation of blood loss remains the most commonly practiced method for quantifying postpartum bleeding; however, it has been shown to be inaccurate, overestimating small volumes of blood loss^6^ and underestimating losses when volumes are high,^7^ thus delaying treatment^8^ and increasing PPH‐related morbidity.^9^ Further, when bleeding is quantified immediately after delivery, cumulative estimation during the first 24 h postpartum is often neglected, leading to missed identification of patients with excessive bleeding.

A review of the evidence in 2023 resulted in two recommendations (WHO recommendations for the prevention and treatment of postpartum hemorrhage).

First, for all women giving birth, routine objective measurement of postpartum blood loss is recommended to improve the detection and prompt treatment of PPH.

Second, a standardized and timely approach to the management of PPH, comprising an objective assessment of blood loss and use of a treatment bundle supported by an implementation strategy, is recommended.

The care bundle for the first‐line treatment of PPH in vaginal births should include rapid institution of uterine massage, administration of an oxytocic agent and tranexamic acid, intravenous fluids, examination of the genital tract, and escalation of care.^10^

Our paper highlights the importance of objective quantification of blood loss during and after childbirth, the various methods available for blood loss measurement, and their application in different clinical settings. Graphic materials and videos are included, which we hope will facilitate the implementation of objective postpartum blood loss quantification strategies, particularly in resource‐limited settings.

## 2 DEFINITION OF POSTPARTUM HEMORRHAGE

There are several published definitions of PPH; however, at the time of writing this paper, no clear consensus exists regarding which is most appropriate, contextually relevant, and effective in initiating timely evidence‐based treatment protocols. A physiologically based definition, focused on hemodynamic and metabolic impact, is likely to correlate closely with clinical outcomes, however typical signs of hypovolemia do not usually appear until approximately 25% of the total blood volume has been lost.^11^ In addition, determination of the exact volume corresponding to that percentage in each patient is challenging, making this definition unreliable for clinical use.

The American College of Obstetricians and Gynecologists (ACOG) defines PPH as a cumulative blood loss of greater than or equal to 1000 mL or blood loss accompanied by signs or symptoms of hypovolemia within 24 h after the birth process.^12^ This definition acknowledges that the mode of delivery (vaginal or cesarean) should not alter the threshold for initiating therapeutic interventions. Furthermore, during a cesarean birth, the patient might receive neuraxial anesthesia, which is associated with a loss of sympathetic tone in certain vascular territories, thereby impairing some of the adaptive mechanisms to blood loss. This might result in a greater hemodynamic impact than what a patient might experience when losing the same volume during a vaginal birth. ACOG emphasizes that, regardless of the mode of delivery, blood loss exceeding 500 mL should raise concerns among the treating team, and depending on the clinical context (e.g. pre‐existing anemia or very low body mass index), it might indicate the need for therapeutic measures.

The current WHO definition includes a lower threshold of 500 mL within the first 24 h after vaginal birth (while aligning with the 1000 mL threshold for cesarean birth),^4^, ^5^ recognizing that in resource‐limited settings (where vaginal birth is the predominant mode of delivery), access to healthcare services is often delayed, and visual estimation of blood loss frequently underestimates the true volume lost.^13^

The continued high prevalence of PPH as a leading cause of maternal morbidity and mortality worldwide, especially in resource‐limited settings, has recently led to expert consensus recommendations advocating for lower diagnostic and treatment thresholds for bleeding during both vaginal and cesarean births.^14^ It has been proposed that during cesarean birth, the first response should be triggered once the woman has lost at least 500 mL of blood with ongoing bleeding or when clinical signs of hemodynamic instability appear, whichever occurs first.^14^

Similarly, the initiation of a PPH treatment bundle could be considered in some women from as little as 300 mL of blood loss.^10^ Indeed, a trigger criterion of 300 mL of quantified postpartum blood loss after vaginal birth plus one abnormal clinical observation (uterine tone, increased bleeding flow, or altered level of consciousness) or an abnormal vital sign was one of the criteria used to initiate interventions in the intervention group of the E‐MOTIVE study, in which a reduction in PPH‐related mortality was demonstrated following early detection and protocolized management.^15^

Other definitions are also valid and might be useful when the treating team cannot accurately quantify blood loss or in specific clinical situations. These include a hematocrit drop of more than 10%,^16^ any blood loss that results in hemodynamic compromise, or significant bleeding in patients with low tolerance to blood loss (e.g. those with severe pre‐existing anemia or cardiovascular conditions).^17^

## 3 IMPORTANCE OF OBJECTIVE QUANTIFICATION OF POSTPARTUM BLOOD LOSS (Box 2)

If the assessment of postpartum blood loss is inaccurate, PPH might go unrecognized, potentially delaying or preventing the timely initiation of life‐saving interventions. In addition, underestimation might lead to suboptimal interventions, when the provider grades the hemorrhage as not severe. Timely recognition of PPH minimizes the risk of life‐threatening complications, such as hemorrhagic shock. Effective interventions, such as the administration of tranexamic acid, can lose their efficacy if administered too late,^18^ likely due to the metabolic deterioration associated with prolonged inadequate oxygen delivery to tissues.

Early identification of abnormal bleeding is one of the key pillars of the intervention bundles proposed by the California Maternal Quality Care Collaborative^19^ and endorsed by ACOG.^17^ Therapeutic interventions used to control abnormal bleeding require time for proper execution, and often by the time they are applied, blood loss far exceeds the thresholds established for diagnosing PPH. It is evident that immediate recognition of abnormal bleeding is fundamental to the successful management of this condition. Changes in maternal vital signs or laboratory parameters often provide late or misleading information.^20^ Visual estimation of blood loss after both vaginal and cesarean birth is notoriously inaccurate^21^ and is thereby of limited clinical use. Accurate and effective assessment of ongoing blood loss, both during and after delivery, is therefore essential for reducing maternal morbidity and mortality.

Evidence has increasingly supported the objective quantification of postpartum blood loss as a fundamental component of safe birth management. The 2018 Cochrane review concluded that there was insufficient evidence to recommend one blood loss quantification strategy over another, as studies found no significant differences in severe morbidity, transfusion rates, or the use of uterotonics when comparing visual estimation with calibrated drapes.^22^ Despite the lack of conclusive evidence favoring a specific method, overall, objective quantification techniques are generally considered to offer more accurate estimates than subjective visual assessments.^23^

Morbidity among women with severe PPH might be reduced when quantitative blood loss estimation is integrated into maternal safety protocols.^22^ Regional experiences in high‐resource countries^24^ as well as in resource‐limited settings^25^ reflect a shift in the weighting of published evidence, now far more clearly recommending a diagnostic approach that combines a calibrated drape for objective blood loss measurement with clinical observations.^26^

BOX 2 **Importance of objective quantification of postpartum blood loss.**

**Table:** 

**Consequences of inaccurate assessment** Visual estimation is often unreliable and underestimates blood loss Delayed or missed diagnosis of PPH increases risk of hemorrhagic shock and mortality Late interventions (e.g. tranexamic acid) may lose efficacy if not administered promptly **Objective quantification saves lives** Accurate measurement enables **timely recognition** and treatment of PPH Early identification is a core element of quality care bundles (e.g. CMQCC, ACOG) Vital signs and labs change late; they are not reliable early indicators **Evidence and practice trends** Cochrane review (2018) found no single superior method, but: ⚬ Objective tools (e.g. calibrated drapes) outperform visual estimation ⚬ Clinical protocols increasingly favor combining quantification + clinical observation **Global implementation** Adoption of quantification strategies is increasing in both high‐ and low‐resource settings Integration into safety protocols may reduce severe PPH‐related morbidity

Abbreviations: ACOG, American College of Obstetricians and Gynecologists; CMQCC, California Maternal Quality Care Collaborative; PPH, postpartum hemorrhage.

## 4 METHODS FOR QUANTIFYING POSTPARTUM BLOOD LOSS

Table 1 describes the different methods for quantifying postpartum bleeding. A commonly used method to assess blood loss during the third stage of labor is visual estimation, typically performed by a trained health worker.^26^ Visual estimation is inaccurate; obstetric caregivers tend to underestimate blood loss by approximately 30%–50%, particularly at higher volumes of blood loss.^7^ This occurs even when the measurement is performed in specialized centers and by personnel with extensive training.^27^ Other factors contributing to the inaccuracy of visual estimation of blood loss include the speed and duration of blood flow and the influence of mode of birth on the health worker's perceptions.

**TABLE 1:** Methods for quantifying postpartum bleeding.

Method		Description	Advantages	Disadvantages
Methods based on direct collection				
Methods based on gravimetry		The use of a scale to weigh absorbent material before and after use. The formula is used: (weight after—weight before)/1.05 = blood volume (in mL; 1.05 g/mL is the density of blood)	High accuracy	Time‐consuming; requires preparation of equipment knowing the “dry weights” of all materials
Volumetric measurement: collection of blood in containers to measure its volume	Blood suction collectors	Method routinely used during cesarean birth. Some collection bags placed under the patient's pelvis can be connected to a negative pressure blood collector	Relatively high accuracy	Attention must be paid to the inclusion of other fluids in the collection containers (urine, cleaning solutions, amniotic fluid)
	Blood collection bags for vaginal birth	Under‐buttock plastic bags that collect blood loss through the vagina	Low cost; can be calibrated and allow real‐time assessment of blood loss volume; can be handmade in each hospital using plastic bags, with marked lines indicating volumes of 300 mL and 500 mL; additional volume marks can be added to facilitate quantification; non‐calibrated bags must be weighed to calculate the fluid volume inside	Lithotomy position required; additional non‐clinical workload for the service provider to prepare the bags with marked lines
	Other commercial blood collection containers	Other low‐cost, reusable containers have been designed with pre‐established volumes that facilitate immediate identification of patients with PPH	Low cost; reusable; lower environmental impact; useful for births in positions other than lithotomy	Not accessible in all countries; unknown even to many doctors
		Other non‐reusable collector devices	Visual analog estimation based on the preset characteristics of the device	Not accessible in all countries
	Artisanal methods for quantifying bleeding	In settings such as home birth or intercultural birth, traditionally accepted fabrics are used by the community during vaginal delivery. These fabrics have a standard size (approximately 100 × 155 cm) and absorb a relatively consistent volume of fluid. Among the names given to this fabric pieces are: “kanga,” “lappa,” and “sarong”	A useful strategy in resource‐limited settings or when the community has low acceptance of medical recommendations; once saturated with blood, these fabrics act as an alarm to trigger hospital transfer or the administration of available and approved medication by traditional healers or midwives	Low accuracy
Visual methods				
Visual estimation		Visual assessment of blood loss volume; a visual reference method is used based on images of known volumes of blood on different surfaces (e.g. gauze or sheets)	Quick; widely used in the world	Low accuracy
Colorimetry cards		Cards with red gradients to compare pad saturation and estimate loss volume	—	Low accuracy; training and graphic material are required to remember the proposed equivalences
Methods based on clinical and paraclinical assessment				
Clinical assessment		Evaluation based on clinical signs and symptoms	No additional equipment required	Subjective and less accurate; clinical signs of hypovolemia are late
Hemoglobin test		Comparison of hemoglobin levels before and after delivery	High accuracy in determining blood loss	Requires blood analysis and laboratory resources; physiologic hemoconcentration during early stages of bleeding may lead to false negative
Advances technological methods				
Spectrophotometry		It allows the measurement of hemoglobin in the collected fluids and to make an accurate quantification of the actual blood volume (discounting other fluids such as amniotic fluid)	Accurate measurement under predetermined conditions	High cost; availability in low‐ to medium‐resource settings is an issue
Applications and sensors		Use of artificial intelligence and sensors to more accurately estimate postpartum bleeding in real time	Automatic calculation; immediate results	High cost; availability in low‐ to medium‐resource settings is an issue

Abbreviation: PPH, postpartum hemorrhage.

Although strategies have been reported to improve the performance of visual blood loss estimation, including staff training and the use of cognitive aids to approximate blood loss, based on the appearance of blood‐soaked surgical materials, the observed improvement is only temporary.^28^

There are other approaches to assess postpartum blood loss after birth more objectively. Implementation of quantitative assessment of blood loss requires the following: (i) use of direct measurement of blood loss (quantitative blood loss); and (ii) clear protocols for collecting and reporting a cumulative record of blood loss after delivery.^19^

Considering the differences between vaginal and cesarean births, we will describe both situations separately.

### 4.1 Vaginal birth

**FIGURE 1:**
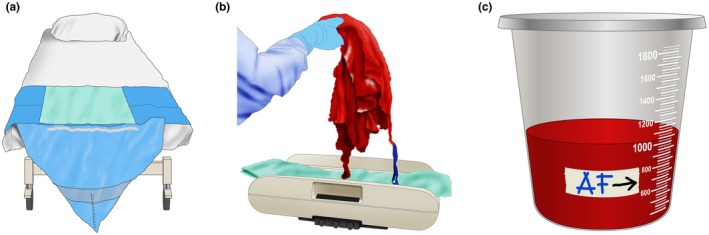
Components of the objective quantification of postpartum bleeding. (a) Graduated under‐buttock plastic bags. (b) Weight of blood‐soaked materials. (c) Fluid level in the suction device after draining the amniotic fluid (AF).

In direct blood collection, a vessel (such as a plastic collecting bag or a tray) is used to collect all blood lost during the third stage of labor^29^ (Figure 1a). To avoid overestimating blood loss due to other fluids (urine, perineal wash solutions, amniotic fluid) that might enter the collection container and considering that in most cases bleeding occurs after placental delivery, placing the device under the maternal perineum immediately before placental removal is recommended. In the case of a collection bag, it can be positioned underneath the woman's buttocks, folded to prevent the collection of other fluids, and then unfolded after delivery of the infant (Video S1).

Graduated collection bags with marked volume indicators (e.g. at 300, 500, and 1000 mL) enable the clinical team to monitor blood loss in real time and to easily recognize when predefined thresholds are exceeded.

**FIGURE 2:**
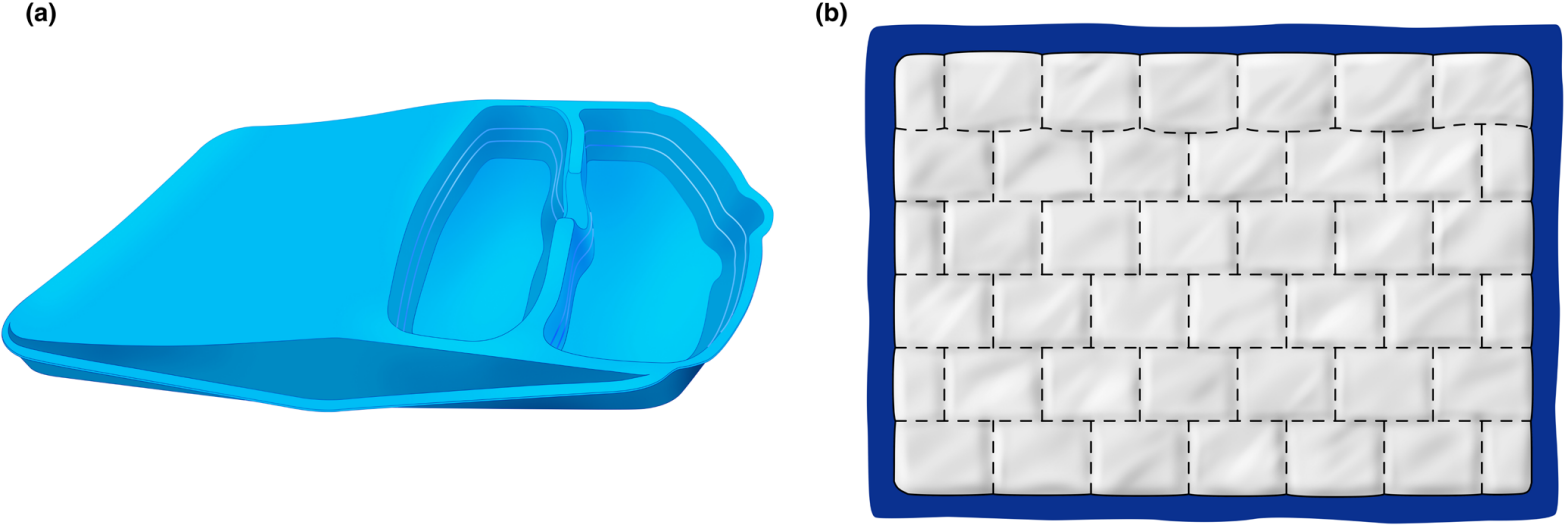
Other devices for postpartum blood collection. (a) MaternaWell Tray. A low‐cost, reusable plastic container designed in South Africa with visual cues corresponding to volumes of 300, 500, and 1000 mL. (b) Signaling a postpartum hemorrhage emergency (SAPHE) mat, designed so that each square absorbs up to 50 mL of blood, allowing for an approximate calculation of the volume lost.

Although plastic bags are relatively inexpensive and can even be locally handmade upon hospital request (Figure 2), maintaining consistent availability in resource‐limited settings might still pose a challenge. A calibrated drape (Dhaka/Dk drape) made with thin, transparent, low‐cost, polythene shopping bags and readily available in the market (capacity 12–15 kg) appears to be effective and safe. The drape might be disposed of, as for other contaminated medical waste. The calculated cost of making the drape is only approximately US$0.05.^30^

**FIGURE 3:**
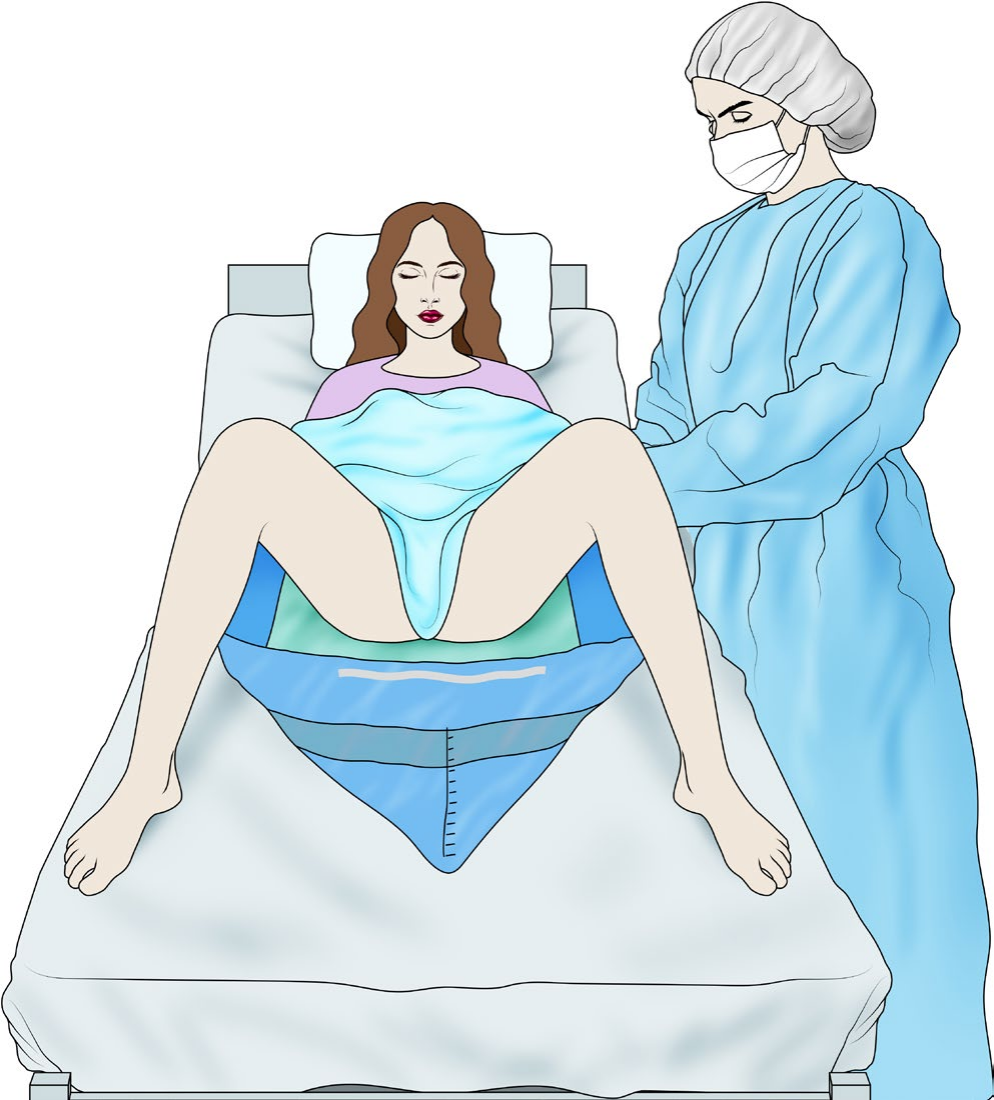
Collection bag in place with the bed tilted. In patients who have experienced postpartum hemorrhage, once they are positioned in the supine position, the collection under the buttock bag can be maintained by Inclining the bed (reverse Trendelenburg position). It is recommended to keep the bag in place for at least 1 h after bleeding control.

Alternatively, non‐graduated plastic drapes can be used. After the bleeding stops, the bag and its contents can be weighed. The team can estimate blood loss using a 1:1 equivalence between grams and milliliters (Video S1). Although there might be economic concerns about adding another item to the standard birth supply kit, as well as environmental concerns related to increased plastic use, these can be addressed through well‐defined disposal protocols. Moreover, the relatively low additional cost is minimal compared to the devastating impact of a PPH‐related maternal death. As an alternative, various models of reusable plastic collection devices are also available with visual cues corresponding to 300‐, 500‐, and 1000‐mL volumes (Figure 3a).

**FIGURE 4:**
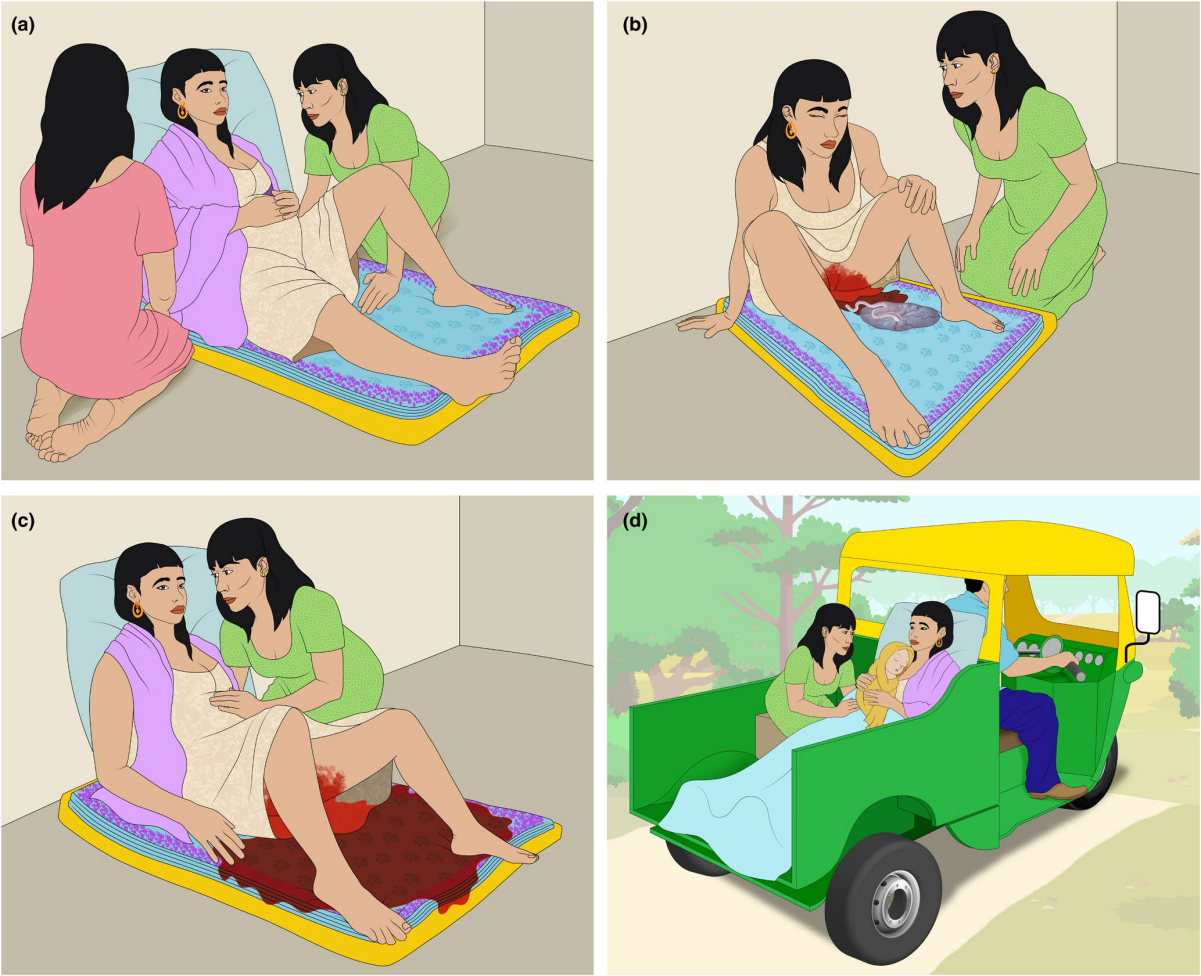
Traditional practices to estimate blood loss during delivery (“Kanga”‐based blood loss estimation). In some communities where home birth is common, traditional healers or midwives use standardized pieces of fabric with an approximate absorption capacity of 500 mL to estimate postpartum blood loss. (a) The laboring woman is positioned over the folded “Kanga,” which absorbs (b) postpartum bleeding. (c) When the entire Kanga is soaked with blood, an alert is triggered. (d) If bleeding continues, the patient must be transferred to the nearest hospital. This approach should be discussed between the hospital covering the patient's residential area and the community members responsible for assisting home births. In such settings, where no other options are available, interventions such as oral misoprostol administration for the active management of the third stage of labor and abnormal postpartum bleeding, manual aortic compression, and hypothermia prevention can be part of the initial management of patients with postpartum hemorrhage (PPH).

These devices are similarly placed under the buttocks after the infant is born, allowing for easy identification of abnormal postpartum bleeding,^31^ with high rates of approval among birth attendants and birthing women themselves.^32^ In cases where the team prefers to keep the collection bag in place for an extended period after birth, simple strategies have been reported to ensure the woman's comfort, such as adjusting the bed position when the bag is in use (Figure 4).

In addition to collecting blood in a container, the attending team should quantify the volume of blood soaked into the surgical clothing used. Surgical sponges, gauze, diapers, sheets, and other blood‐soaked items should all be weighed (Figure 1b). The dry weight of these items must be known and subtracted from the total weight obtained for each case evaluation (Table 2).

**TABLE 2:** Dry weight of surgical clothing and personal hygiene implements used during or after delivery at Fundacion Valle de Lili, Cali, Colombia.

Element	Dry weight (g)^a^
Surgical sponge	21
Gauze	5
Standard surgical drape	52
Patient gown	150
Adult diaper	110
Postpartum sanitary pad	10

^a^ In each hospital, the materials and their sizes vary, and consequently, the volume of absorbed blood differs. Each team must weigh their materials and create a list of “dry weights,” which should be clearly visible to all team members.

**FIGURE 5:**
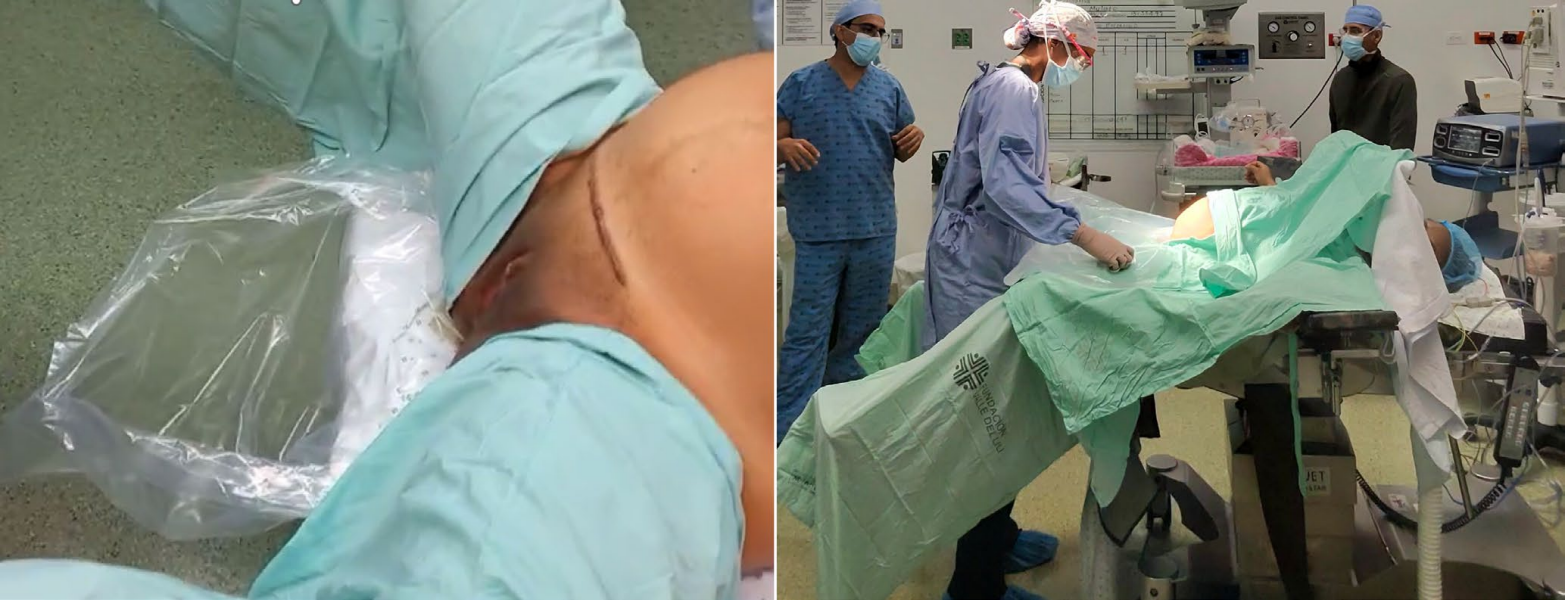
Low lithotomy positioning during cesarean birth for placenta accreta spectrum, allowing the collection bag to be placed under the perineum. Left: The plastic collection bag (graduated or non‐graduated) is placed beneath the patient's pelvis before applying sterile drapes. Lateral stirrups are used to support the lower limbs. Right: The low lithotomy position with the hips abducted, allowing a surgical assistant to be positioned between the thighs and providing a clear view of the perineum through a sterile transparent plastic surgical drape. During the procedure, an assistant periodically checks for vaginal bleeding and monitors the volume of blood accumulated in the collection bag placed beneath the surgical drape.

In resource‐limited settings, other strategies are valid, addressing local needs and incorporating practices accepted by the local community.^33^ For example, during home births, traditional birth attendants use the number of blood‐soaked cloth items (“*kangas*” in Tanzania and “*sarongs*” in Asia) as a threshold measure, after which they administer misoprostol or refer the woman to a health facility.^34^ It has been demonstrated that these cloth items have an average blood absorption capacity of approximately 500 mL (Figure 5).^35^

Other devices have also been developed to collect blood lost vaginally, featuring predefined characteristics that enable a rapid visual estimation of blood loss. An example is the “Signaling a Postpartum Hemorrhage Emergency (SAPHE) mat,” which is composed of superabsorbent polymer, nylon, pellon, and SurgiMat dressings. The mat is designed so that each square absorbs up to 50 mL of blood.^36^ Counting the number of blood‐filled squares allows for an approximate calculation of the volume lost (Figure 3b).

### 4.2 Cesarean birth

**FIGURE 6:**
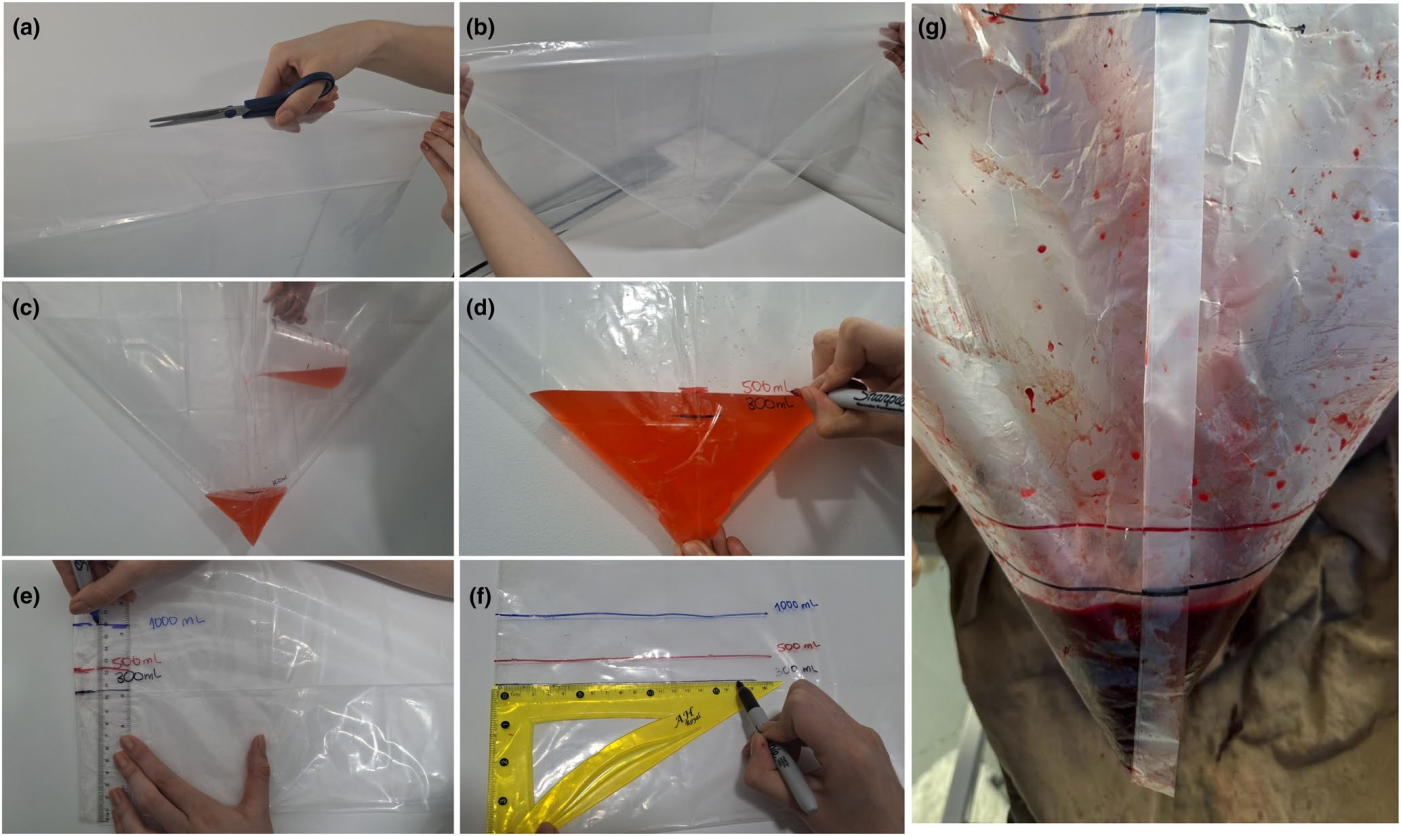
Hand‐made collection bag. By using large, transparent plastic bags, a graduated collection bag can be constructed to quantify postpartum bleeding. (a) One side of the bag is cut and (b) the bag is folded so that it acquires a funnel shape with the closed part facing down. (c) A predefined volume (300 mL, 500 mL, 1000 mL, and other volumes as the local team prefer) is poured in to determine the placement of (d) the alert mark using an indelible marker. (e, f) This marked bag serves as a template to transfer the markings onto other bags without the need to use liquid in each one. (e) For example, a ruler can be used to transfer the markings obtained from the first bag, where the predefined liquid volume was deposited, onto (f) other bags. Additional markings can be added to guide the team in assessing blood loss progression, such as at 300 mL to alert the team (e, f) and at 1000 mL to indicate a higher‐volume hemorrhage. (g) Bags are used during vaginal birth to collect postpartum bleeding. Further calibrations can be made to measure the amount of actual blood loss.

The traditional positioning of a woman during a cesarean birth limits the use of a collection bag under the perineum. In certain situations, such as a cesarean for placenta accreta spectrum (PAS), the use of low lithotomy positioning is recommended, allowing the collection bag to be placed under the perineum (Figure 6). In most cases, however, cesarean births are performed without vaginal bleeding; therefore, blood loss can be estimated more objectively by summing up the weight of the blood‐soaked surgical items (subtracting the dry weight, as in a vaginal birth) and the content of suction devices.

To avoid overestimating blood loss due to amniotic fluid, it is useful for the surgical team to note the fluid level in the suction device after draining the amniotic fluid. At that point, a mark can be placed on the suction device (Figure 1c), and the volume collected after that moment should be considered for quantifying blood loss (Video S2).

There are also other, more complex methods for the measurement of blood loss. However, these can be difficult to perform and are not always available in routine clinical practice (Table 1). This includes hemoglobin concentration change in venous blood sampling comparing pre‐ and postpartum evaluation, spectrophotometry,^37^ dye dilution technique,^38^ contrast‐enhanced ultrasound,^39^ nuclear medicine imaging with the use of radiopharmaceuticals.^29^ New technologies, such as digital analysis of photos of soaked pads and linens, have also been tested.^29^, ^40^ Unfortunately, methods that document blood loss retrospectively are useful for statistical purposes but not for the real‐time blood loss tracking required to trigger effective clinical intervention.

### 4.3 First 24 h after birth blood loss quantification

**FIGURE 7:**
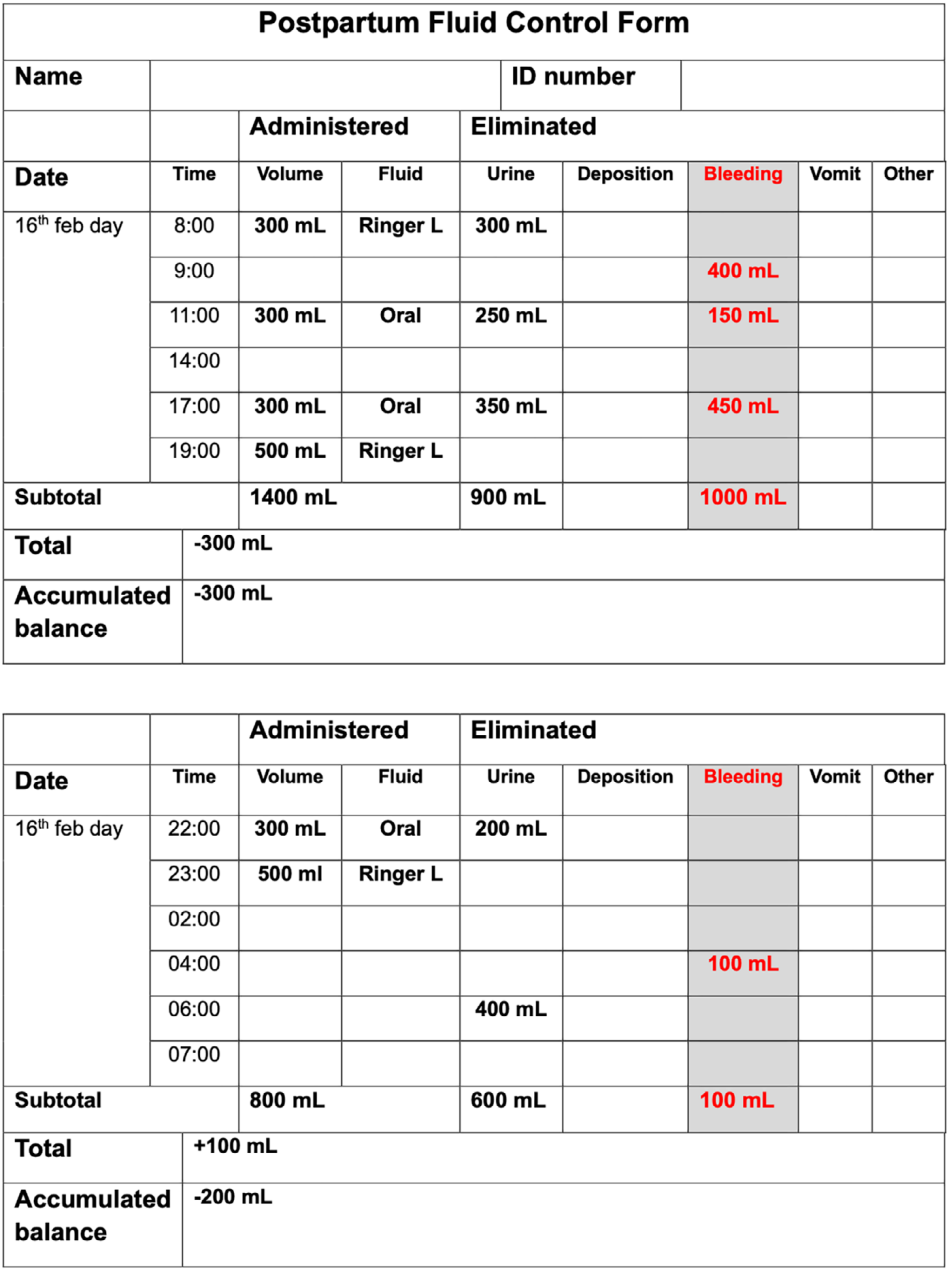
Blood quantification as urine output measurement. Objective quantification of postpartum bleeding should include the documentation of measurements using a format specifically designed to facilitate cumulative tracking over the first 24 h postpartum, similar to the way urinary output is monitored. The definition of postpartum hemorrhage includes the total volume of blood lost during the first 24 h after childbirth.

Current definitions of PPH include not only the volume of blood lost, but also the time frame in which the loss occurs.^11^ Accurate identification of PPH requires objective quantification of blood loss, where possible, over a 24‐h period, not just during the immediate postpartum phase. It is therefore recommended that blood loss be documented in medical records similarly to the way urine output is routinely charted^41^ (Figure 7).

In addition to measuring objective blood loss after birth, immediate postpartum monitoring should, where possible, include the weight of blood‐soaked clothing during the first 24 h. Diapers, sanitary pads, or sheets soaked with blood should be weighed and the attending midwife should subtract the dry weight of these items. The final weight should be converted to milliliters and recorded in the medical record. For example, a patient with a blood loss of 400 mL during vaginal birth is not immediately diagnosed with PPH. However, if a clot weighing 150 g (equivalent to 150 mL) is expelled through the vagina 2 h later and a blood‐soaked diaper weighing 600 g (150 g dry weight of the diaper and 450 g = 450 mL of blood) is removed from that patient 8 h after birth, the diagnosis of PPH can be established because the volume of blood loss exceeds 500 mL (400 mL during delivery + 150 mL 2 h later and 450 mL at 8 h postpartum = 1000 mL lost in the first 24 h after vaginal birth) and immediate treatment must be administered (Figure 7).

As the most widely accepted current definition of PPH is blood loss exceeding 500 or 1000 mL within the first 24 h postpartum, we recommend an objective and cumulative quantification of blood loss at least during this period, unless, or until, the WHO definition changes.

To date, there is insufficient evidence to strongly recommend a specific timeframe for continuing blood loss assessment after diagnosing PPH. However, it appears reasonable to continue ongoing blood loss monitoring as long as active bleeding persists, or, if the patient remains hemodynamically unstable after blood loss exceeding 500 mL,^11^ unless, or until, clear guidance regarding timing is available.

#### Other methods for identifying patients with abnormal postpartum bleeding

**FIGURE 8:**
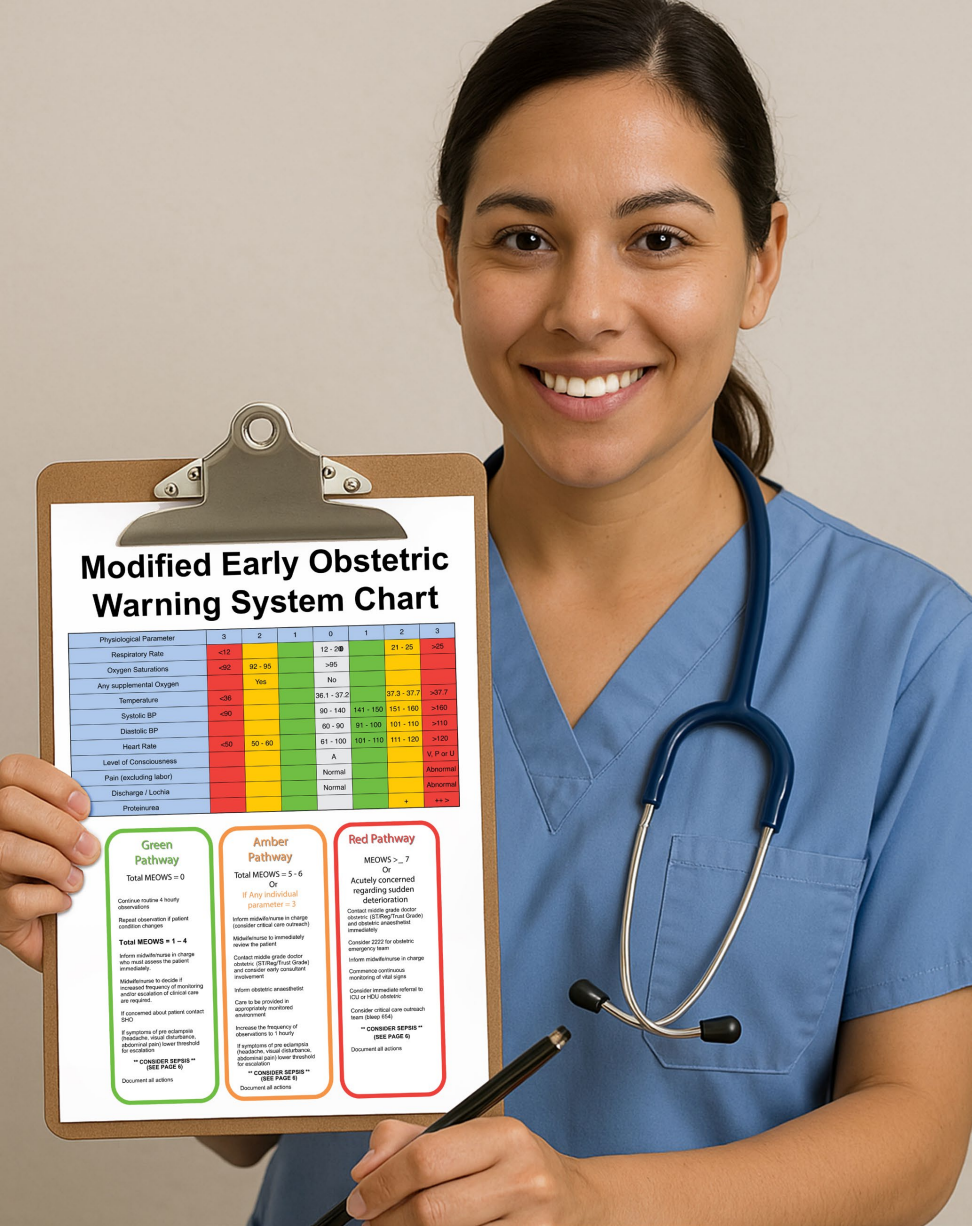
Obstetric early warning system chart. Several models of early warning systems in obstetrics exist, many of which have been scientifically validated. Their routine implementation in obstetric emergency services facilitates the identification of patients at risk of clinical deterioration. These systems are based on the monitoring of physiological parameters and the detection of abnormalities, each assigned a specific score. The cumulative score across all parameters determines the risk of deterioration and guides the need for immediate or delayed interventions.

Despite best efforts to quantify postpartum blood loss, the volume of estimated blood loss remains unreliable in many cases. Therefore, attention should also be directed toward the patient's overall clinical status. This is particularly important in the postoperative period.^23^ Recently, some guidelines have incorporated the shock index (SI; heart rate/systolic blood pressure) and obstetric early warning systems into their recommendations for assessing hemorrhage (Figure 8, Box 3).^42^

BOX 3 **Tips for quantifying postpartum blood loss.**

**Table:** 

**Objective quantification after vaginal birth** Use graduated collection bags or calibrated drapes placed after birth Weigh soaked materials and subtract dry weight (1 g ≈ 1 mL of blood) Low‐cost tools (e.g. SAPHE mat, reusable drapes) are effective and acceptable In home births, counting soaked cloths (e.g. “kangas”) can guide early action **Quantification after cesarean birth** Use weighing of soaked materials and suction device volumes (subtracting amniotic fluid) In some PAS cases, low lithotomy allows for vaginal collection drape placement Mark the suction container after amniotic fluid drainage to estimate blood loss accurately **Quantifying blood loss over 24 h** Cumulative loss must be tracked for the first 24 h postpartum, not just immediately after birth Document soaked items and expelled clots, converting weights to volume Chart blood loss like urine output; intervene when thresholds are exceeded **Clinical tools for PPH detection** SI: pay attention to SI ≥0.9; persistent SI >1 suggests recent significative bleeding Rule of 30: 30% drop in Hb/Hct or BP, HR increase of 30 bpm = ~30% volume loss Vital signs support diagnosis when quantification is not feasible **Implementation considerations** Requires multidisciplinary training and adherence to established protocols A culture shift is needed: from subjective estimation to routine objective quantification Process maps (Table 3) guide implementation for vaginal and cesarean births

Abbreviations: BP, blood pressure; Hb, hemoglobin; Hct, hematocrit; HR, heart rate; PAS, placenta accreta spectrum; SAPHE, signaling a postpartum hemorrhage emergency; SI, shock index.

Any amount of obstetric bleeding that is associated with clinical signs of hypovolemia should prompt a PPH evaluation and consideration of interventional protocols. The SI, along with the “rule of 30,” represents an important set of tools to assist clinicians in emergency situations by estimating blood loss and assessing the degree of hemodynamic instability.

#### Shock index

To date, the normal obstetric SI has been defined as 0.7–0.9, compared to 0.5–0.7 in the non‐pregnant population, considering that the hemodynamic changes of pregnancy might delay the recognition of hypovolemia.^11^, ^43^ To compensate for blood loss, the heart rate increases before a drop in systolic blood pressure, leading to an increase in SI. An SI of 0.9 or higher is associated with increased mortality, and an SI greater than 1 raises the likelihood of requiring a blood transfusion.^44^ FIGO considers the shock index a potential marker of PPH severity and a warning sign of hemodynamic instability when its value exceeds 0.9.^43^

#### Rule of 30

The rule of 30 estimates an approximate blood loss of 30% of normal circulating volume (70 mL/kg in adults, 100 mL/kg during pregnancy). It is defined by a 30% decrease in hematocrit, a 30% reduction in hemoglobin (approximately 3 g/dL), a 30 mmHg drop in systolic blood pressure, and an increase in heart rate by 30 beats per minute.^43^

## 5 IMPLEMENTATION OF OBJECTIVE MEASUREMENT OF POSTPARTUM BLOOD LOSS

Objective strategies for quantifying postpartum bleeding aim to estimate blood loss as accurately as possible, recognizing that absolute accuracy is not possible. Implementing objective measurement requires a coordinated team effort and a cultural shift at an individual and institutional level. This means moving away from a reliance on visual estimation and involving all stakeholders in obstetric care, both in the delivery room and the operating theater. It is essential that the entire team is familiar with and consistently follows established procedures. Table 3 presents process maps for blood loss quantification during vaginal and cesarean births, respectively (Box 4).

BOX 4 **Implementation of objective measurement of postpartum blood loss.**

**Table:** 

**Core strategies for implementation** PPH bundles improve outcomes through coordinated actions: *Readiness*, *Recognition*, *Response*, *Reporting/Learning*, *Respectful Care* Simulation (in‐person and virtual) boosts team readiness, clinical confidence, and cost‐effective training Debriefings support learning, emotional well‐being, and quality improvement **Clinical impact—E‐MOTIVE Trial** E‐MOTIVE (2023): Early PPH detection + treatment bundle reduced severe PPH, surgeries, and deaths Key tools: calibrated drape, response bundle, training, audits, and clinical champions PPH was detected in 93.1% (vs 51.1%) of cases; treatment initiated in 91.2% (vs 19.4%) Reinforces: *early detection is essential to trigger life‐saving care* **Role of professional societies** Scientific societies promote leadership, training, policy, and scale‐up Empowering local champions and inter‐hospital support networks enhances adoption Behavioral change is key: checklists (e.g. BOND) and continuous reinforcement improve adherence **Barriers and enablers** Biggest barrier: resistance to change in clinical practice Environmental concerns (e.g. plastic waste) can be mitigated with biodegradable or reusable drapes Implementation must balance cost, usability, and sustainability, especially in low‐resource settings Virtual mentorship and inter‐institutional alliances boost success in underserved areas **Moving forward** Strong evidence supports routine objective blood loss measurement Adoption may take a decade. Change must be accelerated through strategic behavioral and systemic interventions Hospitals must select context‐appropriate tools and ensure sustainability over time Visual and video materials can support implementation across different clinical settings

Abbreviation: PPH, postpartum hemorrhage.

**TABLE 3:** Process maps for blood loss quantification during vaginal and cesarean birth.

Vaginal birth	Cesarean birth
Gather your team and design a sustainable strategy accepted by all members	
Determine the dry weight of clothing and other items that may become soaked with blood to improve the accuracy of blood loss estimation Create a standardized reference list and pictorial guide with the dry weights of commonly used materials (e.g. surgical drapes, gauze, pads, and kangas) and their estimated blood absorption capacity Ensure this list/guide remains visible to all team members in critical areas such as labor and delivery rooms, operating theaters, and emergency departments	
Train the team on using this method consistently and integrating it into routine PPH management protocols	
Begin blood loss quantification immediately after the birth of the baby and the expulsion of amniotic fluid, before placental delivery	Aspirate and quantify all fluids in the suction device before placental delivery. Mark the suction canister and notify the team of the fluid level
When the bleeding is considered controlled and before adding irrigation fluid, quantify the volume in the suction device and inform the team of the result
Weigh all blood‐soaked materials and clots. Subtract the dry weight of the materials (using the reference list created for each hospital, as weights might vary between institutions) and calculate the blood content using the equivalence 1 g = 1 mL	
At the end of the procedure, sum up the estimated blood loss obtained by weighing materials with the volume collected in the vaginal collection bag or the suction device in cesarean births	
Record the quantified blood loss in the fluid intake and output chart to allow subsequent shift personnel to account for additional blood loss during the first 24 h of postpartum monitoring	
Regularly review and update the strategy to ensure its effectiveness and alignment with best practices	

Abbreviation: PPH, postpartum hemorrhage.

### 5.1 Useful strategies for implementation

The most successful experiences in reducing morbidity and mortality associated with PPH are based on the organization of interdisciplinary teams that implement protocols focused on immediate diagnosis and treatment.^19^, ^24^, ^25^

**FIGURE 9:**
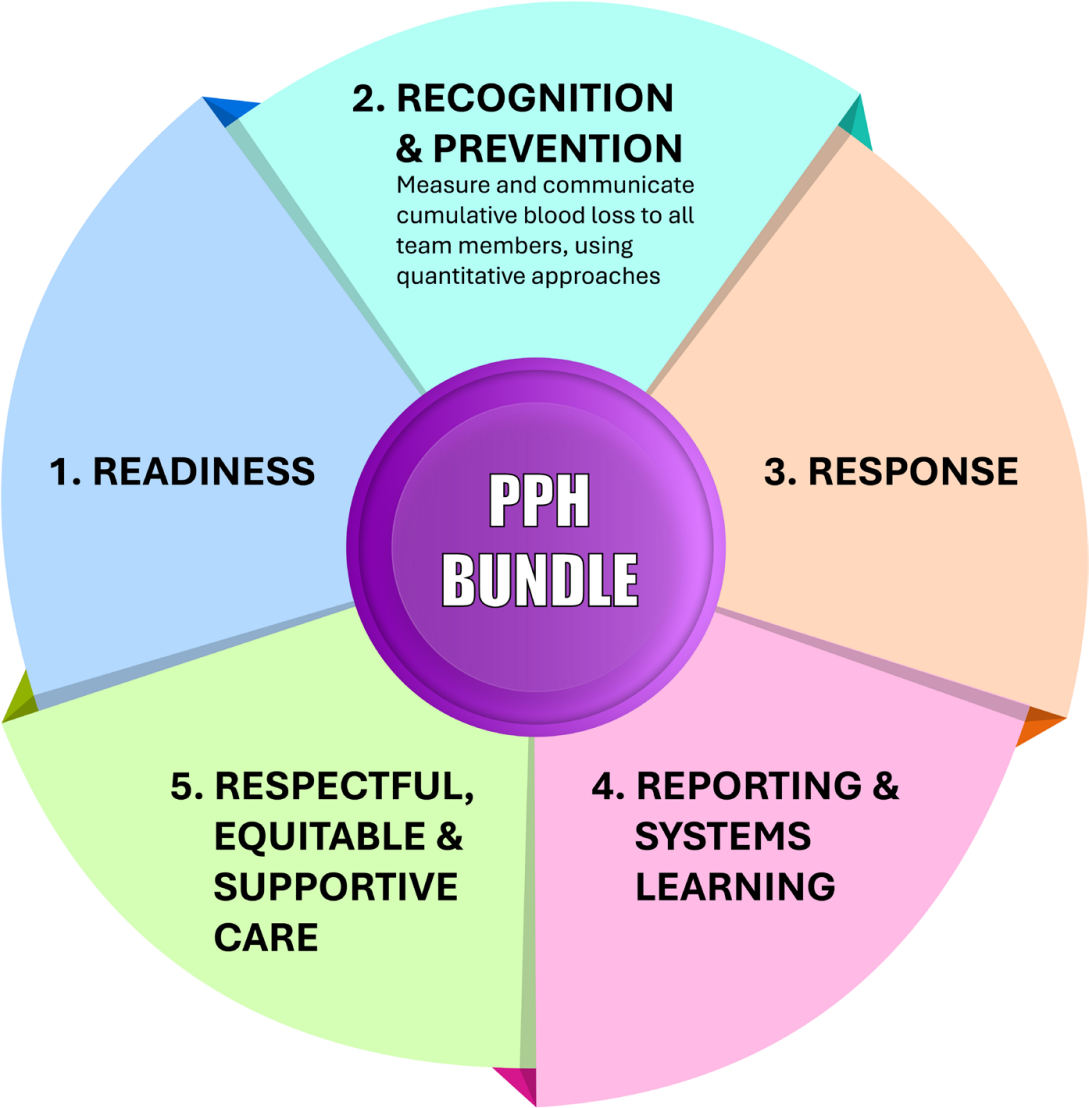
PPH intervention bundle. The organization of activities aimed at improving PPH care includes five established domains, ranging from the preparedness of healthcare systems and professionals (Readiness), through Prevention and early Recognition of the condition, to an optimal, evidence‐based Response. Following the management of the emergency, activities related to Reporting and Systems learning support continuous quality improvement for future cases. The integration of Respectful, equitable, and supportive care policies is essential to ensure a humanized approach to care. One key component of the Recognition domain is the objective quantitative and cumulative measurement of blood loss during the first 24 h postpartum, along with clear communication of the results to the entire care team. Abbreviation: PPH, postpartum hemorrhage.

One of the most widely accepted models is the use of intervention bundles (Figure 9), which recognize that an appropriate response to a patient with PPH requires prior preparation of the team and hospital, as well as early identification of abnormal bleeding. These three activities (Readiness, Recognition, Response) can only be progressively improved if they are complemented by continuous audit of results, team feedback, and quality improvement in care (Reporting/System learning).^10^, ^19^ Recently, a fifth component has been added to this bundle—Respectful, Equitable & Supportive Care—which encourages open, empathetic, and transparent communication among members of the multidisciplinary team, including the patient and their support network.^45^

Training and continuous updating of the team is essential for implementation. Simulation training for PPH, including virtual simulation, significantly improves competency of health service providers by improving knowledge, skill, and attitude, resulting in improvement of clinical performance.

Enhancing team spirit and team response times in a safe and controlled environment for healthcare providers to practice identification and management of PPH helps develop confidence in handling life‐threatening emergencies. It is a cost‐effective way to provide high‐quality training to a large number of healthcare providers, compared to traditional methods, and can be used to continuously update the existing and new workforce. Virtual obstetric simulation training is widely considered to be feasible, acceptable, and effective.^46^, ^47^, ^48^

Debriefing after a PPH is a crucial process for learning, performance improvement, and reducing potential psychological trauma of the staff team. It involves a structured discussion immediately after an event, to analyze what went well, and what could be improved, which helps improve management of future cases. It is also used as a checklist during emergencies, including near‐miss cases.^49^, ^50^

Although the evidence‐based technical knowledge required to manage a case of PPH has been well‐documented in the scientific literature for many years, the real challenge lies in adapting this knowledge to diverse settings, ensuring provider adherence, and sustaining its application over time, despite considerable challenges. Nearly 10 years ago, the implementation of bundles in regional hospital systems and individual hospitals reported reductions in severe maternal morbidity among patients who experienced PPH after bundle implementation.^25^, ^51^, ^52^ More recently, the application of a similar intervention bundle in Africa confirmed its effectiveness in reducing maternal deaths due to PPH.^25^

The PPH emergency response using the bundle approach has clinical components. These are the first response bundle (uterine message, oxytocic, intravenous fluid, and tranexamic acid along with supportive measure) and refractory bundle (compressions, intrauterine tamponade, anti‐shock garments, necessary surgery, blood/blood product transfusion, and proper referral). Non‐clinical components include facility readiness, network integration, teamwork and communication, respectful maternity care, data, monitoring, quality improvement, and leadership. Professional member societies can play a significant role in implementation, advocacy, and scale‐up.^53^

It is impossible to determine whether these improvements are due to specific practices within the bundle or to the implementation of the bundle as a whole; however, early identification of PPH patients is very likely to be a key factor in improving outcomes, particularly when implementing other interventions requires additional time.

### 5.2 Clinical impact of blood loss quantification

In May 2023, the findings of the E‐MOTIVE trial were published.^25^ This was a cluster randomized trial on early detection and a PPH treatment care bundle involving 80 hospitals. The intervention consisted of a calibrated drape for early PPH detection, followed by a first‐response PPH treatment bundle. The intervention also included the use of implementation strategies (audit and feedback, health worker training, PPH trolley, carry case, and change champions). The control group received postpartum blood loss collection in uncalibrated drapes (without alert or action lines) to quantify blood loss for the study, and their PPH treatment interventions were administered according to usual care practices.

In the intervention arm, early detection of PPH included the following: a calibrated blood collection drape was used to collect all postpartum blood and to prompt health workers to treat PPH when excessive blood loss occurred. The drape was applied for 1 h for all women after vaginal birth. For women with excessive bleeding, the drape remained in place for an additional 2 h from the point of excessive bleeding detection. Health workers received training on the use of a blood loss monitoring chart to document blood volume loss, blood flow, and uterine tone every 15 min in the first hour postpartum. Blood pressure and pulse were checked and documented at least once in the first hour postpartum.

Postpartum hemorrhage was detected in 93.1% of patients in the intervention group compared to 51.1% in the usual‐care group (rate ratio = 1.58; 95% confidence interval [CI] = 1.41–1.76), and the treatment bundle was used in 91.2% and 19.4%, respectively (rate ratio = 4.94; 95% CI = 3.88–6.28). The study concluded that early detection of PPH and the use of a bundled treatment approach led to a lower risk of severe PPH, laparotomy for bleeding, or death from bleeding than usual care among patients having a vaginal birth.

The E‐MOTIVE trial illustrates how the organized use of strategies, previously described as effective in PPH treatment, can improve clinical outcomes in resource‐limited settings. Interventions such as uterine compression, administration of uterotonic medications, and intravenous fluids are well known to all healthcare professionals involved in childbirth care. Moreover, this study crucially highlights the importance of immediate detection of abnormal bleeding (in this case, using a calibrated drape) as the trigger for these interventions.

### 5.3 The role of scientific societies and academic groups

Adherence to the recommendation for objective blood loss measurement also depends on non‐behavioral interventions,^54^ which were emphasized in the E‐MOTIVE trial.^25^

In all these successful experiences incorporating objective quantification of blood loss, collaboration between hospitals and/or academic groups played a critical role. Hospitals that began using blood quantification devices received training in these processes and mid‐term follow‐up from a central support group. The identification and empowerment of local champions, who sustained initial momentum and mobilized their teams over time, was a determining factor.

One model of implementation can be seen in the “Leadership Development Initiative: Removing barriers to Effective Access and Coverage of maternal Healthcare (LDI:REACH),” a FIGO‐delivered program that facilitates leadership development and quality improvement in clinical practice, aiming to effect change in healthcare systems. Its long‐term goal is to improve maternal and newborn health outcomes through increased coverage of key clinical interventions, rapidly implemented at scale, through the leadership of National Member Societies, all conducted through the lens of gender diversity and equity.

FIGO's member societies are critical to improving maternal and newborn health outcomes within their respective countries. They actively participate in national legislative policy guideline development, providing technical assistance, capacity building, educating communities, and conducting implementation research.

Many member societies not only draw on obstetricians and gynecologists but also include key health professionals, such as midwives and general practitioners. One of the four clinical areas FIGO LDI:REACH has focused on, is E‐MOTIVE for PPH.^55^

It is not always easy to change ingrained behaviors among healthcare workers. Medium‐ to long‐term interventions, such as reminders, local leadership promotion, positive feedback, and indicator measurement, are essential.^25^, ^54^, ^56^ Surgical checklists have been shown to promote the implementation of safe practices.^57^ One example of a checklist to promote implementation of blood loss measurement during third stage care is the mnemonic “BOND”: Baby skin‐to‐skin; Oxytocin; iNitiate blood loss monitoring; and Delay cord clamping.^58^

Routine objective quantification of blood loss during and after childbirth represents a structural change in obstetric care, addressing the challenges, including manufacturing and standardization costs, supply, transportation, and training on blood collection device usage. One study examined the cost of introducing quantitative postpartum blood loss assessment compared to visual estimation, considering the direct costs of resources such as the calibrated drape, transportation, staffing, additional training, periodic refresher training, equipment, supervision, and monitoring,^59^ concluding that the measure is associated with significant savings of resources.

### 5.4 Barriers to implementation

Concerns have been raised regarding the environmental impact of plastic drapes, as well as the safe disposal of these materials in areas with a high prevalence of human immunodeficiency virus. These concerns are conflicting: discarding the collection device after a single use and avoiding washing and reuse reduces the bio‐sanitary risk, whereas reusing it decreases the environmental impact. In response, cheaper, reusable, environmentally friendly,^31^ and easier‐to‐use options (Figure 3a) have been developed, as well as “in‐hospital” handmade alternatives^30^ (Figure 2) that can help mitigate cost‐related challenges. The concern of environmental hazards can be mitigated by biodegradable transparent materials made from jute and corn flour.^30^ It is the responsibility of professional societies and government organizations to facilitate access to these devices in resource‐limited settings.

Interestingly, according to one report, the most significant barrier to adopting objective blood loss quantification as a management standard for all births is the resistance to change among healthcare professionals.^60^

There is now clear and sufficient evidence to strongly recommend this practice^25^ and to incorporate it into the educational content provided to all healthcare professionals involved in maternity care. It is essential to recognize that the widespread adoption of evidence‐based interventions can take more than a decade.^61^ The decision to accelerate the implementation of objective blood loss quantification in all births will require the use of innovative, validated strategies that serve as change accelerators. Behavioral interventions and sustained inter‐institutional collaboration at all levels have been associated with protocol adherence and reduced maternal mortality due to PPH.

Recently, the incorporation of virtual communication and mentorship programs, where resource‐limited hospitals are supported by more experienced institutions, has demonstrated positive outcomes in improving obstetric emergency management.^62^

Although a single specific strategy for objective postpartum blood loss quantification has not yet been defined, the minimum requirements for an accessible measurement device in resource‐limited settings (where most PPH‐associated morbidity occurs) have been outlined (Videos S1–, S3), highlighting that this strategy must be low cost, easy to use and interpret in different scenarios, and ideally environmentally friendly.^63^

For now, each hospital must define which strategy (e.g. graduated drape, non‐graduated drape, or reusable plastic container) is most applicable to its local context, facilitating easy, sustainable implementation and long‐term adherence to this early PPH detection strategy.

We hope that the graphic content of this article will be helpful for the implementation of objective blood loss measurement methods during vaginal and cesarean births.

## 6 CONCLUSION

There is an urgent need to implement objective quantification of postpartum blood loss as a standard practice in maternity care. Early detection, combined with timely therapeutic interventions, is critical to reducing the global burden of PPH and associated maternal morbidity and mortality. Clinical trials, such as E‐MOTIVE, have demonstrated the effectiveness of bundled approaches that include calibrated blood loss measurement, provider training, and structured response protocols.

To ensure sustainability and widespread adoption, these practices must be formally integrated into training curricula for all levels of maternal healthcare providers, incorporated into institutional guidelines, and supported by national health policies. The shift from visual estimation to objective quantification represents a fundamental change in clinical culture and will require coordinated behavioral, structural, and policy‐level interventions to overcome resistance, address resource constraints, and adapt tools to diverse care environments.

Global implementation must prioritize context‐appropriate strategies, including the use of reusable or biodegradable drapes, virtual simulation training, and inter‐institutional support networks. Only through a systemic and collaborative approach can we achieve lasting improvements in the timely recognition and effective treatment of postpartum hemorrhage worldwide.

## AUTHOR CONTRIBUTIONS

Article conceptualization: FB, AJN‐C, DS. Literature review or critical analysis of the topic: FB, AJN‐C, DS. Manuscript writing—original draft: AJN‐C. Critical review and editing of the manuscript: FB, AJN‐C, DS, JH, JP‐J, AB, MAS, JMB‐L, JB‐K, AEU, AW. Viewing or preparing graphs/figures: FB, AJN‐C, DS. All authors read and approved the final manuscript.

## FUNDING STATEMENT

No funding was received.

## CONFLICT OF INTEREST STATEMENT

The authors have no conflicts of interest to declare.

## Supporting information


**Video S1.** Objective quantification of blood loss after vaginal birth.

**Video S2.** Objective quantification of blood loss after cesarean birth.

**Video S3.** Making low‐cost under‐buttock calibrated drapes with plastic bags.

## Data Availability

Data sharing is not applicable to this article as no new data were created or analyzed in this study.
